# Development of Flexible and Partly Water-Soluble Binder Systems for Metal Fused Filament Fabrication (MF^3^) of Ti-6Al-4V Parts

**DOI:** 10.3390/polym16172548

**Published:** 2024-09-09

**Authors:** Ralf Eickhoff, Steffen Antusch, Dorit Nötzel, Marcel Probst, Thomas Hanemann

**Affiliations:** 1 Institute for Applied Materials, Karlsruhe Institute of Technology, Hermann-von-Helmholtz-Platz 1, 76344 Eggenstein-Leopoldshafen, Germany; steffen.antusch@kit.edu (S.A.); dorit.noetzel@kit.edu (D.N.); marcel.probst@student.kit.edu (M.P.); or thomas.hanemann@imtek.uni-freiburg.de (T.H.); 2 Department of Microsystems Engineering, University Freiburg, Georges-Koehler-Allee 102, 79110 Freiburg, Germany

**Keywords:** 3D printing, additive manufacturing, FFF, FDM, MF^3^, MEX, titanium alloys, Ti6Al4V, water-soluble binder, flexible filaments

## Abstract

Metal Fused Filament Fabrication provides a simple and cost-efficient way to produce dense metal parts with a homogenous microstructure. However, current limitations include the use of hazardous and expensive organic solvents during debinding for flexible filaments the stiffness of filaments made from partly water-soluble binder systems. In this study, the influence of various additives on different partly water-soluble binder systems, with regard to the flexibility and properties of the final parts, was investigated. Furthermore, a method using dynamic mechanical analysis to quantify the flexibility of filaments was introduced and successfully applied. For the first time, it was possible to produce flexible, partly water-soluble filaments with 60 vol.% solid content, which allowed the 3D printing of complex small and large parts with a high level of detail. After sintering, density values of up to 98.9% of theoretical density were achieved, which is significantly higher than those obtained with existing binder systems.

## 1. Introduction

Additive manufacturing of metals is dominated by Selective Laser Melting (SLM) and Electron Beam Melting (EBM) [1,2,3,4]. In recent years, Metal Fused Filament Fabrication (MF^3^), also known as material extrusion (MEX) or Fused Filament Fabrication (FFF), has established itself as a potential substitute, mainly due to its accessibility and low overall costs. Especially for small production volumes, MF^3^ offers considerable advantages due to its low investment costs and, consequently, low costs per part [5]. Additive manufacturing of medical implants is a growing field [6]. The fabrication of implants adapted to the patient at low costs can improve their medical treatment and therefore, improve the patient’s quality of life. Nevertheless, there are still obstacles that need to be addressed. For instance, very large and thin parts, in combination with support structures, can present challenges [7]. Another advantage of MEX is the wide range of materials that can be processed. Apart from the metals mentioned, the production of ceramic parts using FFF has also come into focus in recent years. There are several reports of printed structural ceramics [8,9,10,11]. There are also reports of printed functional ceramics for cathodes of lithium-ion batteries [12,13] and for entire solid-state batteries [14]. Another field of interest is the combined printing of metals and ceramics to enhance the properties of the final parts [15]. However, co-sintering poses a challenge due to differing coefficients of thermal expansion of each material therefore, it requires further investigation [15].

To obtain dense and defect-free parts, production must follow a process chain derived from powder injection molding [16,17,18]. It can be divided into five steps: (1) raw material characterization, selection, and compounding; (2) extrusion of filaments; (3) printing of components using MEX; (4) debinding; and (5) heat treatment. First, the metal powders and polymers are characterized, including powder particle size distribution, density, particle morphology, specific surface area, differential scanning calorimetry, and thermo-gravimetric analysis. Then, suitable combinations of metallic powders and polymeric binders must be selected. The binder usually consists of a high-viscosity backbone polymer, a soluble and low molecular weight base polymer, and various additives. The proportion of each component depends on the desired rheological properties of the later-produced filaments. The metal powder and the polymeric binder are compounded into a homogeneous feedstock, which generally contains a solid content of around 60 vol.%. The produced feedstock is then pelletized and extruded into a filament, which is then wound onto a spool. 3D printing can be conducted on cost-effective, commercial 3D MEX printers. Some modifications are made to improve print quality, for instance, a new extruder for a better grip of the filament [11,19,20], or an alternative heating bed surface to prevent warping [21]. After shaping, the binder must be removed. This can be accomplished in three ways: thermal debinding, combined solvent and thermal debinding, and catalytic debinding. Thermal debinding can cause defects and take more time [22,23], catalytic debinding is limited to select filler materials and binders [23]. Therefore, the combined solvent and thermal debinding process have been established as an alternative. For the first step, the use of organic solvents is common due to the frequent use of non-polar base polymers [24,25,26,27]. Partly water-soluble binder systems, which represent a more economical, more sustainable, and less hazardous approach by using water as a solvent, are almost exclusively found in powder injection molding [28,29,30]. Only one study was found where partially water-soluble binder systems were used for FFF. However, the filaments did not show a flexible behavior [31]. To the best of our knowledge, the fabrication of metal or ceramic parts via MEX with flexible, partly water-soluble filaments, has not been published. A final sintering step in a vacuum or argon atmosphere densifies the metal and controls its structure and mechanical properties. Titanium shows a high affinity for oxygen, nitrogen, and carbon, which leads to the formation of oxides, nitrides, and carbides and in return, increased tensile strength but decreased elongation [32]. Binder systems with a high oxygen content in particular can cause problems [31]. However, contamination during the process chain is generally low and remains below the standards for medical implants [31,33].

This study aims to develop flexible and partly water-soluble binder systems that enable good usability and allow the manufacturing of defect-free titanium parts with a high level of detail.

## 2. Materials and Methods

### 2.1. Material Selection

For potential healthcare applications such as implants, the present work uses a Ti-6Al-4V alloy powder (Grade 23, Tekna Advanced Materials Inc., Sherbrooke, QC, Canada). Due to the demanding mechanical properties of surgical implants, this grade was selected because of its low carbon and oxygen content, which is important to meet the standard for Ti-6Al-4V surgical implants (ASTM F2885-17 [34]). The specifications were derived from surgical implants produced by metal injection molding (MIM). To predict and control the rheological properties of the feedstocks, the particle density via Helium pycnometry (AccuPyc II 1340, Micromeritics Instr. Corp., Norcross, GA, USA), the specific surface area via the BET method (Gemini VII 2390, Micromeritics Instr. Corp., Norcross, GA, USA), and the particle size distribution via laser diffraction (LA-950 Horiba Ltd., Kyoto, Japan) were measured.

Thermoplastic binder systems consist of at least three components, including one or two high-viscosity backbone polymers, a low molecular weight base polymer, and an additive that serves as a plasticizer and a surfactant. In this work, three different polar binder systems, consisting of water-soluble polyethylene glycol (PEG 8000 and PEG 20,000, C. Roth GmbH Co. KG, Karlsruhe, Germany), and the backbone polymers poly(vinyl butyral) (PVB Mowital 30H, Kuraray Europe GmbH, Frankfurt, Germany) and poly(methyl methacrylate) (PMMA Degalan G7E, Roehm GmbH, Darmstadt, Germany), were investigated. In addition, the influence of three plasticizers on each composition was researched. Stearic acid (SA, C. Roth GmbH Co. KG, Karlsruhe, Germany) is a common additive in powder injection molding, while the two shorter fatty acids, capric acid (CA, Sigma Aldrich, St. Louis, MO, USA), and lauric acid (LA, Sigma Aldrich, St. Louis, MO, USA), showed potential for improving the flexibility of the feedstocks in preliminary tests. The plasticizer PAT-77/P (E. & P. Wuertz GmbH & Co. KG, Bingen, Germany), which is commercially available for metal feedstocks, was recommended by the vendor. The plasticizer concentration was calculated as a percentage by weight of the total binder and subtracted from the base polymer PEG. According to previous studies [31], the mixture of PMMA and stearic acid as well as PVB and PAT-77/P yielded less convincing results due to binder separation. These combinations have therefore been omitted. All investigated binder compositions are described in Table 1. The exact compositions of the feedstocks are shown in the respective sections. In addition to the manufacturer’s specifications, the thermal behavior of the polymers and additives was characterized by TGA-DSC measurements (Netzsch STA 449 F3 Jupiter, Netzsch GmbH & Co. KG, Selb, Germany). The density was measured using the same device as the metal powder.

### 2.2. Compounding and Extrusion

Compounding the feedstocks was performed on a torque recording mixer-kneader (W50-EHT, Brabender GmbH, Duisburg, Germany) for one hour at a blade rotating speed of 30 rpm. This corresponds to a shear rate between 2 and 36 s^−1^ [35]. Through the different glass transition temperatures of the backbone polymers, the compounding temperature varied between 110 °C (PVB/PEG), 160 °C (PMMA/PVB/PEG), and 170 °C (PMMA/PEG). Due to inhomogeneities observed in previous studies [36], smaller PMMA granules and a higher rotational speed of 60 rpm were used for some feedstocks. The smaller PMMA granules were produced by extrusion with a capillary rheometer (Rheograph 25, Goettfert Werkstoff-Pruefmaschinen GmbH, Buchen, Germany) and subsequent pelletizing with an impact mill (Granulator 1514, Rapid Germany, Kleinostheim, Germany). The use of the smaller PMMA granules is detailed in the respective section. The feedstocks were then extruded into filaments with a diameter of 2.85 mm by a single screw filament extruder (Noztek pro HT, Noztek, Shoreham, UK). The corresponding extrusion temperature for each feedstock is outlined found in the respective section. If the feedstocks showed sufficient flexible behavior, the filaments were wound onto a spool with a diameter of 20 cm using a winder (Noztek Filament Winder 1.0, Noztek, Shoreham, UK). Otherwise, the filaments were extruded onto a metal cooling track and cut off at 50 cm.

### 2.3. Rheological Characterization

The rheological properties of the feedstocks were investigated using two methods. On the one hand, the shear rate-dependent flow behavior of the feedstocks was characterized using a high-pressure capillary rheometer (Rheograph 25, Goettfert Werkstoff-Pruefmaschinen GmbH, Buchen, Germany). The following parameters were applied:

- Temperature: 160 °C,
- Capillary length and diameter: 30 and 1 mm,
- Shear rate range: 1 to 3000 s^−1^.

On the other hand, to characterize the flexibility of the filaments, the temperature-dependent viscoelastic behavior was measured by oscillatory measurements in the temperature sweep (TS) mode by a dynamic mechanical analyzer (DMA 242 E Artemis, Netzsch GmbH & Co. KG, Selb, Germany) equipped with a dual cantilever sample holder (2 × 16 mm free bending length). The following parameters were utilized:

- Temperature: 20–50 °C,
- Frequency: 0.5 Hz,
- Amplitude: 50 µm,
- Pre-force: 0.1 N.

The temperature range and a low frequency of 0.5 Hz were selected to simulate the handling of the filament by a human operator.

### 2.4. Fused Filament Fabrication

A commercial, but modified MEX printer (x350pro, German RepRap, Feldkirchen, Germany) was used for printing. Instead of the original extruder, an E3D Titan Extruder and E3D V6 Hotend 2.85 mm filaments were installed (E3D-Online Ltd., Oxfordshire, UK). In addition, the print bed was covered with PE-coated spring steel to ensure enhanced adhesion of the parts during printing. For a higher level of detail in the printed parts, a more powerful surface ventilation was installed. Slicing was performed with an Ultimaker Cura (V 5.4.0). Ten cubic samples of each feedstock were produced for subsequent characterization. The dimensions are described in [21]. All samples were printed with a layer height of 0.1 mm, a rectilinear pattern, and an infill angle offset of 90° per layer. As different researchers have assessed, gaps between the extruded round lines can decrease the density of the printed parts [37,38,39,40]. Therefore, the infill density was increased to 105% and the infill overlap was set to 0.2 mm. Additional parameters including temperature or print speed are detailed in their respective sections.

### 2.5. Debinding and Sintering

A two-step debinding procedure was conducted to reduce thermal debinding time and debinding-related defects. First, the PEG was removed in water for 24 h and at 20 °C (PVB/PEG), 30 °C (PMMA/PVB/PEG), or 40 °C (PMMA/PEG) to create open pores. As warm water can induce cracks within the printed parts, water temperature was lowered for PVB-based feedstocks [41]. Then, the parts were dried overnight in a vacuum drying oven at 40 °C to remove the residual water. Thermal debinding was conducted before sintering in a metal sinter furnace (MUT Advanced Heating GmbH, Jena, Germany) using a heating rate of 1.5 °C/min and a maximum debinding temperature of 550 °C. Sintering was subsequently conducted with a heating rate of 5 °C/min and a maximum temperature of 1350 °C. Both heat treatments were performed under an argon atmosphere to avoid contamination with oxygen and nitrogen.

### 2.6. Characterization of Sintered Samples

The printed and sintered samples were characterized using the Archimedes principle (Secura 225D-1S equipped with YDK 01, Sartorius Lab Instruments GmbH & Co., KG, Göttingen, Germany). For relative density calculations, the pycnometer density of the Ti-6Al-4V powder was used as a basis. CT images for pore distribution and defect analysis were conducted on a GE phoenix v|tome|x s240 (General Electric, Boston, MA, USA).

## 3. Results and Discussion

### 3.1. Materials Characterization

To achieve high densities of the printed parts during sintering, a high solid loading of the metal powder in the feedstocks is necessary. However, this leads to increased viscosity, which can prevent the filling of the voids between the extruded round lines during printing. Therefore, a solid load of 60 vol.% was selected for all feedstock systems to reconcile both objectives. Table 2 provides an overview of the measured particle properties. The powder showed a narrow monomodal particle size distribution with an average particle size of approximately 28.6 µm. In addition, a density of 4.4 g/cm^3^ and a specific surface area of 0.15 m^2^/g (SSA) was measured. Furthermore, a spherical morphology of the particle was observed, which results in favorable flow properties.

Knowledge of the density and thermal properties of the polymers used is essential to correctly establish the composition of the feedstocks and the parameters of the compounding process. Furthermore, these characteristics play an important role during printing and thermal debinding. Therefore, all used components were characterized by TGA-DSC measurements. Table 3 lists the measured values for density, softening temperature, and decomposition temperature. The latter were derived from the TGA-DSC measurements. The list also contains the average molecular weight of the polymers as it is an important parameter for the rheological properties and thus for the subsequent feedstocks.

### 3.2. PVB/PEG

As mentioned in previous studies [31], PVB/PEG-binder systems are used in injection molding and FFF, especially in combination with ceramic fillers. To date, there are no reports on flexible filaments with this binder system. Therefore, three different additives with varied contents were used to achieve flexible filaments with high usability and strong properties of the sintered parts. The ratio of 50 vol.% of the backbone polymer PVB to 50 vol.% of the base polymer PEG 20,000 remained the same for all feedstocks.

#### 3.2.1. Compounding and Extrusion

**Figure 1:**
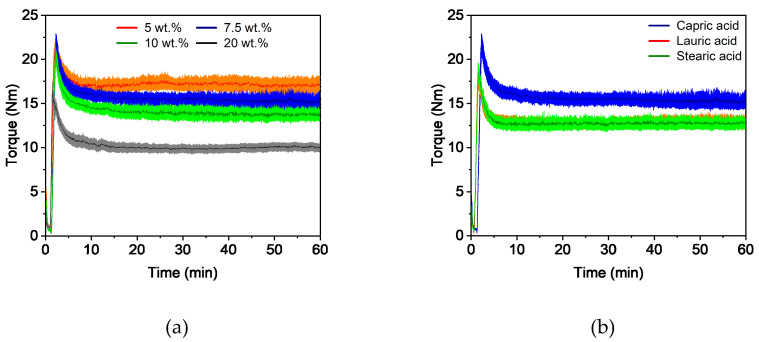
Compounding of PVB/PEG-based binder (110 °C) with different compositions: (**a**) variation of capric acid content and (**b**) variation of the fatty acids with 10 wt.% additive.

In general, the compounding curve can be divided into three phases: the filling phase, mixing phase, and equilibrium phase [42]. Figure 1a shows the torque curves for four different capric acid contents. The overall compounding behavior was the same for all additive proportions and this corresponded to the already described theory. A short mixing phase was followed by a long equilibrium phase of approximately 50 min with an almost constant torque, which indicates a homogeneous feedstock [43]. As expected, the torque decreased with increasing additive content. This is due to the short chain length of the additive, which lowers entanglement between the polymer chains. It also acts as a lubricant between the polymer chains, reducing inner friction and allowing the chains to glide. Besides the additive content, the additive type also has an influence on the compounding curve (Figure 1b). While feedstocks with lauric acid and stearic acid exhibited nearly the same behavior, the addition of capric acid increased the torque slightly.

After compounding, the feedstocks were extruded using the Noztek filament extruder, equipped with a 2.8 mm nozzle. The extrusion temperatures are listed in Table 4. All filament diameters were close to 2.9 mm and thus, they were suitable for the modified x350pro printer. From the produced filaments, only the feedstocks with 10 and 20 wt.% capric acid and 20 wt.% lauric acid was flexible enough to be wound onto a spool.

#### 3.2.2. Rheological Characterization

##### Capillary Rheology

**Figure 2:**
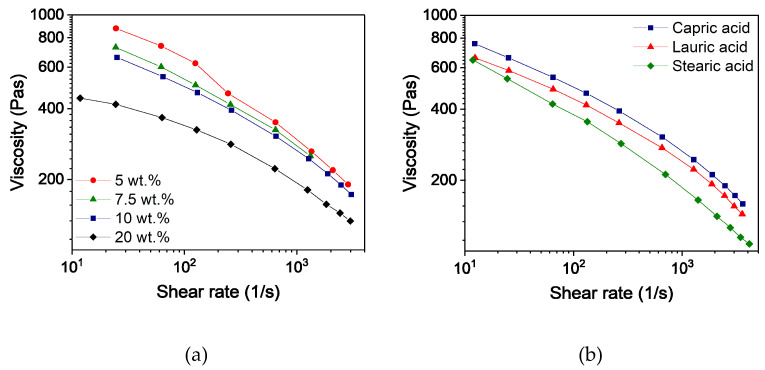
Capillary rheology of PVB/PEG based binder at 160 °C with different compositions; (**a**) Variation of capric acid content and (**b**) variation of the fatty acids with 10 wt.% plasticizers.

To assess and compare the feedstocks, knowledge about the rheological behavior as a function of the composition is crucial. Due to the shear-thinning behavior of the filled polymers, the viscosity is dependent on the shear rate. According to Beran et al. [44], the shear rate in the nozzle can be calculated and is influenced by the layer height, the printing speed, and the inner diameter of the nozzle. The selected printing parameters (layer height 0.1 mm, printing speed 8 mm/s, and inner nozzle diameter 0.4 mm) resulted in a shear rate of 40 s^−1^. In comparison to other shaping methods like injection molding, this value is comparatively small. Figure 2a shows the shear rate-dependent viscosities of the feedstocks with different capric acid contents. As observed during the compounding process, the viscosity dropped with increasing additive content due to less entanglement and the effect of a lubricant. Differences in viscosity between the three additives were small but can be seen in Figure 2b. Furthermore, a slight separation of PEG and PVB could be observed in feedstocks with less than 10 wt.% capric acid and lauric acid as well as in all feedstocks with stearic acid.

##### DMA

**Figure 3:**
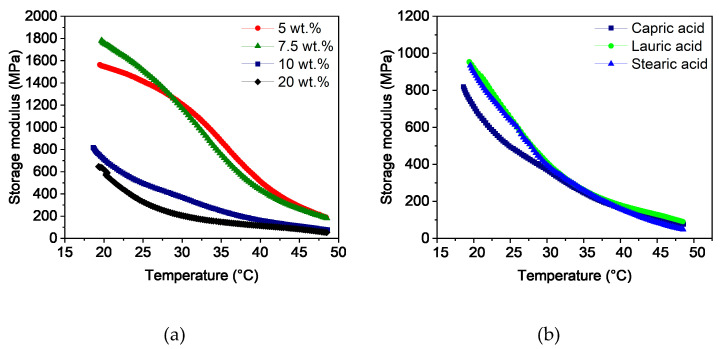
Temperature sweeps of PVB/PEG-based binder with different compositions: (**a**) variation of capric acid content and (**b**) variation of the fatty acids with 10 wt.% plasticizers.

To determine the flexibility of the filaments at room temperature, temperature sweeps were carried out in a range from 20 to 50 °C. Figure 3a shows the storage modulus as a function of the capric acid content. As expected, the storage modulus decreased with increasing temperature due to the higher polymer chain movement. Furthermore, the influence of the additive can be seen. While the rigid filaments with 5 and 7.5 wt.% capric acid had high values of around 1400 MPa at 25 °C, the windable filaments with 10 wt.% and more additive content were below 500 MPa. At higher temperatures of around 50 °C, the softening of PVB and PEG dominated the viscoelastic behavior. As a result, the properties of the feedstocks converged. The influence of 10 wt.% lauric and stearic acid on the storage modulus compared to capric acid can be seen in Figure 3b. At 25 °C, the filaments with 10 wt.% lauric and stearic acid content had slightly higher values of around 650 to 700 MPa than the filaments with capric acid. This behavior is likely a result of the smaller size of capric acid, which can act as a lubricant between the polymer chains even at low temperatures. Since these two filaments could not be wound onto a spool, it was apparent that a value below 500 MPa at 25 °C is required for sufficient flexibility. For even better handling, the storage modulus should have a value of around 400 MPa at 25 °C.

#### 3.2.3. Fused Filament Fabrication

**Figure 4:**
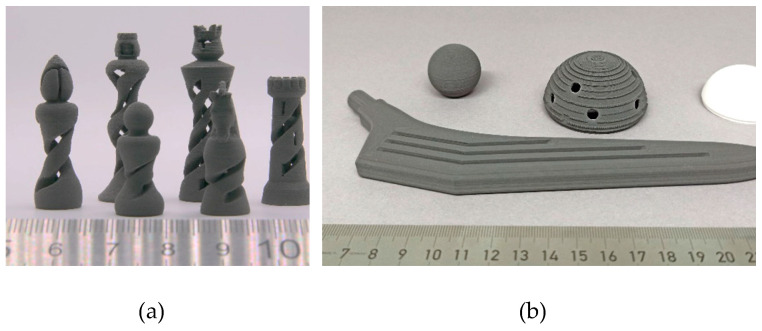
(**a**) Printed chess figures (7.5 wt.% lauric acid) and (**b**) printed hip endoprosthesis (5 wt.% lauric acid) with polymer inlay.

As shown in the previous section, almost all filaments were too rigid to be wound onto a spool. Therefore, the filament rods were fed into the extruder one by one. An exception included the two feedstocks with 20 wt.% capric and lauric acid. Although these filaments presented good flexibility, they were too soft and consequently were ground by the gear of the extruder. For this reason, no samples could be printed from these two feedstocks. The same problem was observed to a lesser extent with the filaments containing 10 wt.% capric and lauric acid. Table 5 lists the investigated printing parameters for the used feedstocks. An advantage of the PVB-based feedstock is the rapid solidification during cooling, which makes the feedstocks suitable for complex geometries with overhangs (Figure 4a). Interestingly, the use of the part surface ventilation led to clogging of the nozzle and was therefore omitted. However, due to the stiffness of the filaments, printing larger parts (Figure 4b) by refilling the filament rods into the printer was not very user-friendly.

#### 3.2.4. Debinding and Sintering

**Figure 5:**
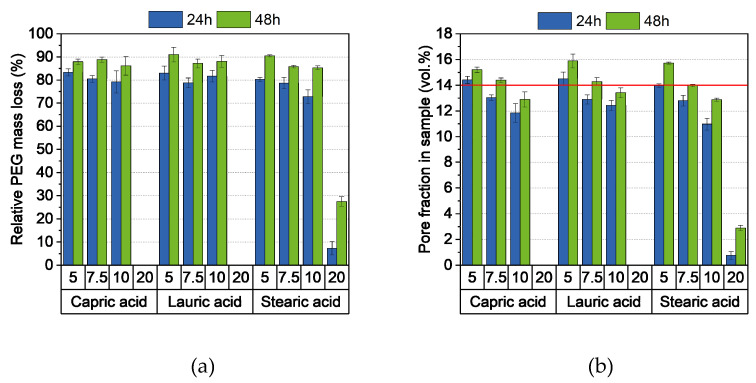
(**a**) Relative PEG mass loss and (**b**) pore fraction in samples after 24 and 48 h debinding in water at 20 °C. 14 vol.%. Pore fraction is a strong first indicator of whether a sample can be thermally debinded and sintered without defects.

Debinding was performed in a two-step process: First, the PEG was removed in water at 20 °C. Figure 5a shows the PEG removal as a function of the type and quantity of additive. Approximately 80% of the PEG was eliminated after 24 h, with the proportion decreasing slightly as the amount of additive increased. However, this difference, together with a slight improvement in PEG removal, could be corrected by further extending the exposure time to 48 h. An exception was the feedstock with 20 wt.% stearic acid. In this system, only a small amount of PEG was removed, which is possibly due to the low PEG content in the feedstock itself. As a result, not enough pores were created to transport the PEG out of the sample. Due to the varying content of PEG in the feedstocks, the significance of the removed PEG content is limited. Therefore, the proportion of pores created in the samples is a more accurate indicator of whether a sample can be thermally debinded without defects. As expected, Figure 5b shows a decreasing proportion of pores with increased additive content. shows that a pore content of 14% by volume is a strong first indicator of defect-free thermal debinding with the chosen heating rate. However, the low green body strength during debinding in water was striking, which was due to the high water absorption of PVB [45]. As a result, complex or large geometries such as the chess pieces or the hip implant depicted in Figure 4 showed deformations.

**Figure 6:**
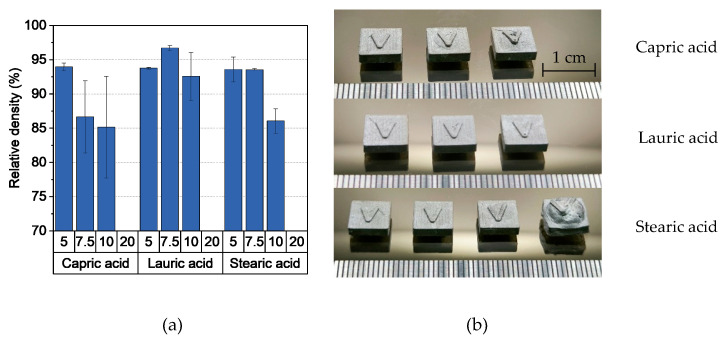
(**a**) Relative density of the samples after sintering and (**b**) sintered samples (from left to right: 5 wt.% to 20 wt.% additive).

After solvent debinding, the printed parts were thermally debinded followed by a sintering step. Figure 6a shows the achieved relative density values depending on the additive content. For the samples containing 5 wt.% additive, a consistent theoretical density of around 93.5% can be observed. With 7.5 wt.% additive content, the density varies significantly between the different additives. This is possibly due to two countervailing effects: on the one hand, the additional additive content lowers the viscosity of the feedstock, which leads to a better filling of the voids between the extruded lines. On the other hand, a higher additive content results in lower porosity, which can prevent defect-free thermal debinding. With 10 wt.% and higher additive content, defects, in particular, blistering, were observed for every sample (Figure 6b). No density could be measured for samples of the feedstock with 20 wt.% stearic acid, as the samples stuck firmly to the sintering base, which would have falsified the density measurement significantly.

### 3.3. PMMA/PEG

PMMA/PEG systems are well-known as binders in powder injection molding of metals and ceramics. Therefore, we focused on the rheological properties at high shear rates and the debinding behavior in water [28,29,30,46,47]. However, only a few cases are reported in the literature in which this binder is used for printing, where the stability and flexibility of the filaments were not the focus [31,48]. As with the PVB-based feedstocks, the influence of three different additives with varied contents on the rheological behavior and the subsequent properties of the printed and sintered samples was investigated. Due to the long chain length of the PMMA molecules and the resulting high viscosity, the ratio for all feedstocks was 40 vol.% PMMA to 60 vol.% PEG 8000.

#### 3.3.1. Compounding and Extrusion

**Figure 7:**
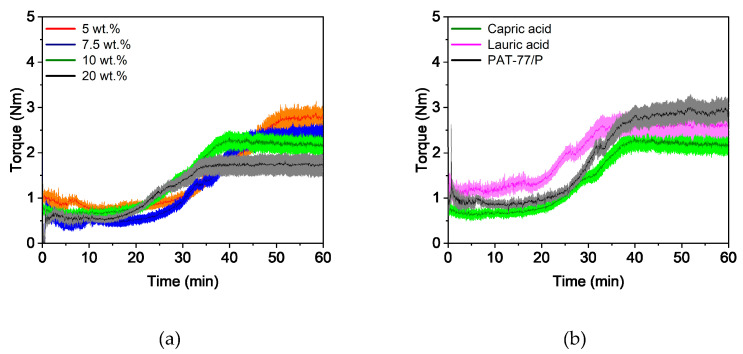
Compounding of PMMA/PEG-based binder (170 °C) with different compositions: (**a**) variation of capric acid content, and (**b**) variation of the plasticizers with 10 wt.% plasticizer.

In comparison to PVB-based feedstocks, compounding was carried out at a higher temperature (170 °C) due to the high softening temperature of PMMA. Figure 7a shows the influence of the capric acid content on the compounding behavior. An increase in the additive content of these feedstocks also led to a drop in the torque curves. However, the differences between the torque curves were quite small. Compared to the PVB-based feedstocks, the y-axis had to be enlarged fivefold to determine the differences between the curves. This is likely due to the high molecular weight of PMMA, which causes strong entanglement and high inner friction between the polymer chains. Therefore, it dominates the rheological properties of the feedstocks. Furthermore, the torque curves deviate from the ones described in the literature, especially in the equilibrium phase. After approximately 20 min, the torque increased drastically, which was also observed in [36]. This behavior can be attributed to the size of the PMMA pellets, which were approximately 3 mm, so they required some time to liquefy. In Figure 7b the influence of the various additives is depicted. No significant differences were found, but feedstocks with capric acid showed the lowest torque compared to PVB-based feedstocks.

The produced feedstocks were then extruded into filaments. The parameters used are listed in Table 6. Surprisingly, the extrusion temperatures were low and were below the glass temperatures of PMMA (109 °C) and PEG 8000 (65 °C). In these cases, the additives acted as a lubricant between the polymer chains and the extruder wall and thus reduced the softening point of the binder. PEG 8000 was presumably responsible for this behavior in the PAT-77/P-based feedstocks. Also striking was the smooth surface of the filament containing 20 wt.% PAT-77/P. This is likely due to the high content of PAT-77/P and the high extrusion temperature, which can lead to deposition of PAT-77/P on the surface. Nevertheless, all filament diameters were close to 2.9 mm and thus suitable for printing. Furthermore, the filaments with 7.5, 10, and 20 wt.% capric acid and 20 wt.% lauric acid could be wound onto a spool.

#### 3.3.2. Rheological Characterization

##### Capillary Rheology

**Figure 8:**
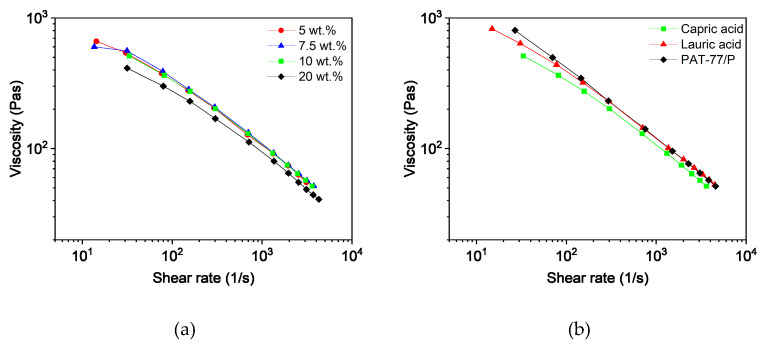
Capillary rheology of PMMA/PEG-based binder at 160 °C with different compositions: (**a**) variation of capric acid content, and (**b**) variation of the fatty acids with 10 wt.% plasticizer.

As previously indicated in the torque curves during compounding, the influence of the additive content and additive type is limited (Figure 8). The long polymer chains of PMMA dominate the flow properties, with only high additive contents leading to a slight drop in viscosity. Despite the higher PEG content and the use of a lower molecular weight, the viscosity remains similar to that of the PVB-based feedstocks. All feedstocks again exhibited shear-thinning behavior.

##### DMA

**Figure 9:**
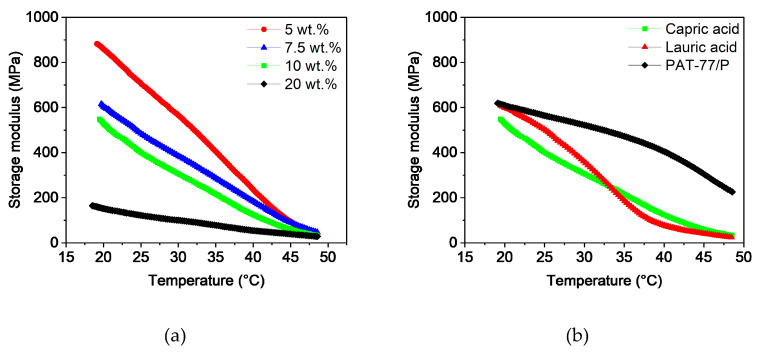
Temperature sweeps of PMMA/PEG-based binder with different compositions: (**a**) variation of capric acid content, and (**b**) variation of the fatty acids with 10 wt.% plasticizer.

The storage modulus was again used to characterize the flexibility of the filaments. Figure 9a shows the influence of the capric acid content on the storage modulus. Unlike the PVB-based feedstocks, the filaments had lower overall storage modulus values. This is likely due to the higher backbone polymer content and the strong hydrogen bridges in the PVB-based feedstocks. In addition, the additive content, in contrast to the shear viscosity, had a significant impact on the flexibility of the filaments. Even with 7.5 wt.% capric acid, the filaments showed a sufficient flexibility (storage modulus values below 500 MPa at 25 °C). However, a high usability could only be observed for the filament with 20 wt.% capric acid. On the contrary, the filaments with 10 wt.% lauric acid and PAT-77/P show higher storage modulus values and therefore, lower flexibility (Figure 9b). Flexible filaments could be manufactured only with 20 wt.% lauric acid (Figure 9b). Moreover, the behavior of the feedstocks with PAT-77/P is notable; all filaments have approximately the same storage modulus values (between 535 and 565 MPa at 25 °C). An explanation for this cannot be provided due to the unknown composition of the additive.

#### 3.3.3. Fused Filament Fabrication

As previously mentioned for the PVB-based feedstocks, the stiff filament rods had to be fed into the extruder one by one. This circumstance reduces user-friendliness of the system and hampers printing larger components. Furthermore, a high content of 20 wt.% capric and lauric acid decreased the hardness of the filaments, causing the filaments to be ground by the gear in the extruder. As a result, it was not possible to print these two feedstocks Table 7 gives an overview of the investigated printing parameters: no significant differences in the processing between the feedstocks could be observed. It is only noticeable that the printing temperature is slightly above the decomposition temperature of capric acid. However, this only affects a short period of time and has no negative impact on the printing result. In contrast to the PVB-based feedstocks, the samples showed a low green body stability above 40 °C, which can influence the printing quality of complex structures. In particular, structures with large differences in dimensions and overhangs can cause problems. In this case, strong part cooling during printing is necessary to achieve a high level of detail. However, it was possible to use a nozzle of 0.15 mm in diameter with these feedstocks, despite the ratio between this nozzle size and the filler diameter being 5. For this low ratio, nozzle clogging could be observed in previous studies [37,44].

#### 3.3.4. Debinding and Sintering

**Figure 10:**
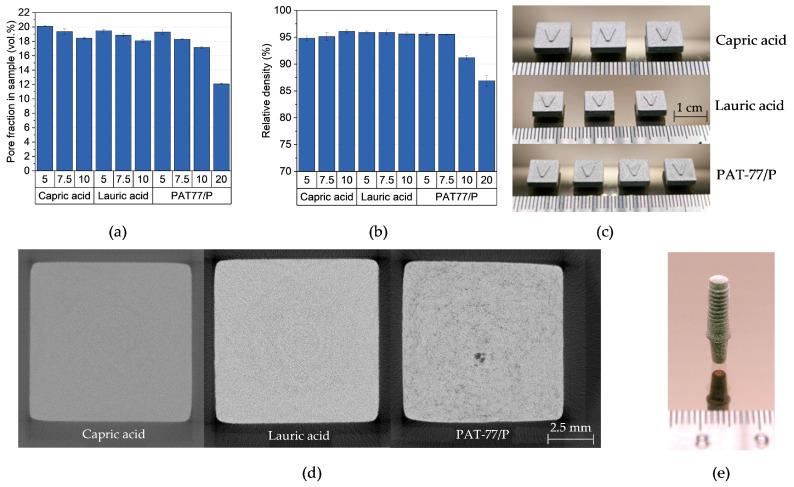
(**a**) Pore fraction in samples after 24 h debinding in water at 40 °C, (**b**) relative density after sintering, (**c**) sintered samples (from left to right: 5 wt.% to 20 wt.% additives), (**d**) CT images of sintered samples with a 10 wt.% additive, and (**e**) sintered dental implant (5 wt.% PAT-77/P).

Due to the high content of PEG in the feedstocks, the samples showed a high pore proportion of 18 to 20 vol.% after solvent debinding (Figure 10a). As expected, this fraction decreased with increasing non-water-soluble additive content. For parts containing 20 wt.% PAT-77/P, this value decreased to 12 vol.%. This is the result of the low PEG content combined with the high amount of non-water-soluble additive PAT-77/P, which acts as a barrier and reduces the diffusion of the PEG molecules. However, in contrast to the PVB-based feedstocks, no deformation was observed, which is likely due to the high water resistance of PMMA. The consequences of the low debinding degree can be observed in Figure 10b and 10c; the low pore fraction led to the formation of pores in the samples, which reduced the density significantly. Surprisingly, the samples containing 10 wt.% PAT-77/P also showed a decreased relative density, although no defects could be observed on the surface. However, as can be seen in Figure 10d, a lot of pores can be observed in the sample. This is the result of the high decomposition temperature of PAT-77/P, which is in the same range as the PMMA used. In contrast to the fatty acids, which combust at lower temperatures and therefore create pores in the sample, too much gas was released which led to the formation of small blisters in the sample. The relative densities of the other feedstocks showed similar behaviors and were in the range of 95.5 and 96.1%. Nevertheless, the implant in Figure 10e validates the ability of the PMMA-based feedstocks to manufacture parts with a high level of detail.

### 3.4. PMMA/PVB/PEG

As previously indicated, both binder combinations (PVB/PEG and PMMA/PEG) have unique properties. While good printing behavior with rapid solidification was observed for PVB-based feedstocks, PMMA-based feedstocks showed strong hardness of the filament even with a low proportion of the backbone polymer and a high green body strength during solvent debinding. Therefore, the two binder systems were combined to utilize their advantages. First, preliminary tests of a binder composition of 15 vol.% PMMA, 35 vol.% PVB, and 50 vol.% PEG 8000 showed a promising combination of both desired properties. Capric acid, lauric acid, and stearic acid were used as additives in proportions of 10 wt.%, 12.5 wt.%, and 15 wt.%, respectively.

#### 3.4.1. Compounding and Extrusion

**Figure 11:**
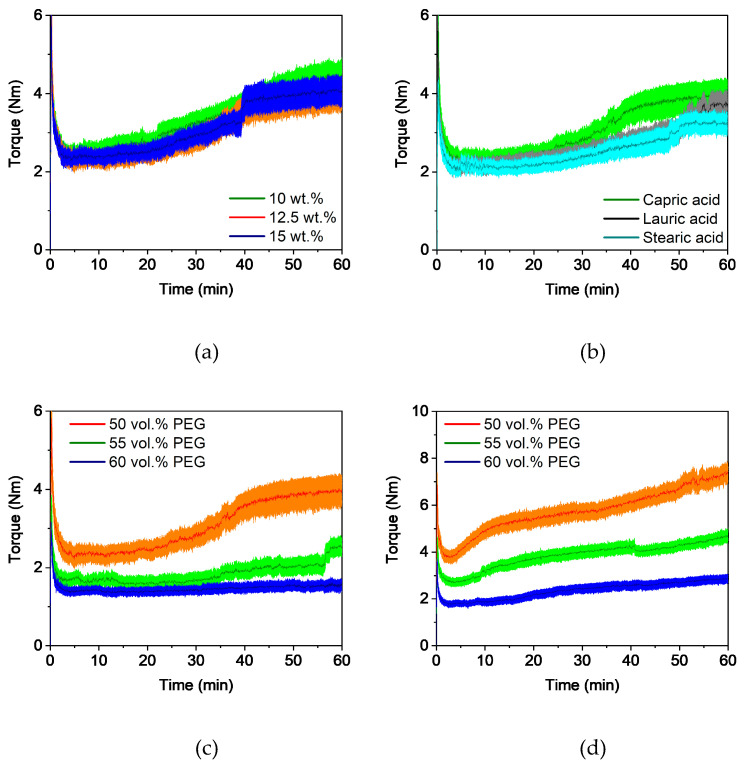
Compounding of PMMA/PVB/PEG-based binder (160 °C) with different compositions; (**a**) capric acid, (**b**) comparison with 12.5 wt.% additive, (**c**) capric acid (12.5 wt.%) with different PEG contents, and (**d**) capric acid (12.5 wt.%) with different PEG contents at a rotational speed of 60 1/s and smaller PMMA granules.

Due to the lower PMMA content in the feedstocks, the compounding temperature was decreased to 160 °C compared to the pure PMMA feedstocks. Figure 11a shows the influence of the capric acid content on the compounding behavior. It can be seen that no significant differences were found. Despite the low proportion of 15 vol.% of PMMA, it still dominated the compounding behavior. Liquefaction of the PMMA pellets could still be observed but with a lower increase in torque. Furthermore, significant differences between the three additives could not be observed. The liquefaction of PMMA began 10 min earlier with capric acid (Figure 11b). Due to the high additive content at the expense of PEG, solvent debinding and the subsequent thermal debinding can cause challenges. Therefore, two feedstocks with a higher PEG proportion were produced. To avoid compromising the hardness of the filaments, the proportion of PVB was reduced. Due to the high PEG content, the viscosity of the feedstock was too low at 60 vol.% PEG and in some cases at 55 vol.% PEG, so much so that the PMMA granules could not be liquefied. This is shown by the torque curve, where almost no increase could be observed (Figure 11c). In addition, non-liquified PMMA pellets were found in the feedstocks after compounding. As previously mentioned, smaller PMMA granules were used and the rotational speed of the blades was doubled, which resulted in a shear rate twice as high [35]. Due to these modifications, liquefaction shifted to an earlier time and increased the torque for all three feedstocks (Figure 11d). In addition, no constant torque was observed after a one hour compounding time.

After compounding, the feedstocks were extruded into filaments with the parameters specified in Table 8 The extrusion temperatures were below the softening point of PMMA. While all filaments containing stearic acid were stiff, filaments with capric acid and lauric acid were flexible enough to be wound onto a spool. In particular, the filaments with 12.5 wt.% demonstrated a good compromise between flexibility and hardness.

#### 3.4.2. Rheological Characterization

##### Capillary Rheology

**Figure 12:**
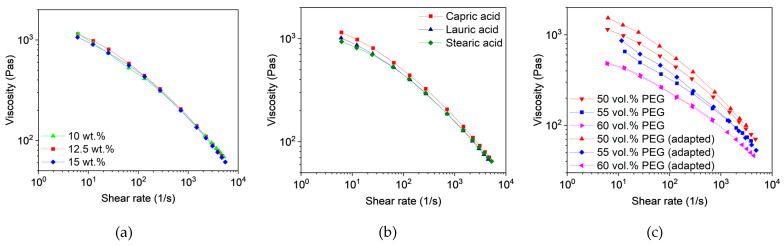
Capillary rheology of the PMMA/PEG-based binder at 160 °C with different compositions; (**a**) variation of capric acid content, (**b**) variation of the fatty acids with 12.5 wt.% plasticizer, and (**c**) capric acid (12.5 wt.%) with different PEG proportions and the adapted feedstocks, which were produced with smaller PMMA granules and a higher shear rate.

The results of the rheological characterization correspond with the findings during compounding. Neither the additive content (Figure 12a) nor the additive type (Figure 12b) showed a significant influence on the flow properties of the feedstocks. However, a higher proportion of PEG at the expense of PVB led to a decrease in viscosity (Figure 12c). While the rheological behavior of the low molecular PEG 8000 and the small additives is almost the same, the replacement of PVB resulted in less entanglement and fewer hydrogen bonds between the polymer chains. Interestingly, the non-liquified PMMA pellets had no impact on the flow behavior. Only for feedstock with 50 vol.% PEG and 12.5 wt.% capric acid did the modifications lead to slightly higher viscosity.

##### DMA

**Figure 13:**
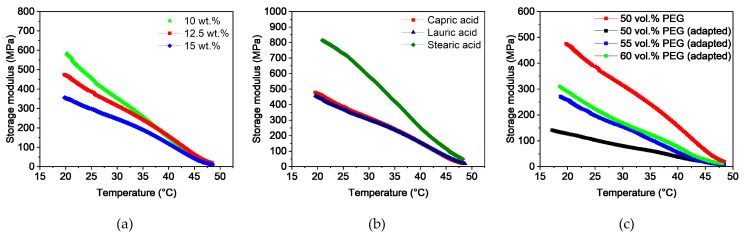
Temperature sweeps of the PMMA/PEG-based binder with different compositions; (**a**) variation of capric acid content, (**b**) variation of the fatty acids with 12.5 wt.% plasticizer, and (**c**) capric acid (12.5 wt.%) with different PEG proportions and the adapted feedstocks, which were produced with smaller PMMA granules and a higher shear rate.

In contrast to the shear viscosity, the additive content (Figure 13a) as well as the additive type (Figure 13b) have a significant influence on the flexibility of the filaments. The higher the additive content, the lower is the storage modulus in the relevant temperature range of 20 to 25 °C. While filaments with 10 wt.% capric acid content have sufficient flexibility, filaments with 12.5 and 15 wt.% guarantee better handling. The same behavior can be observed for lauric acid. On the contrary, filaments with stearic acid show a brittle behavior, which is likely due to the longer stearic acid molecules and thus, less chain movement. Two interesting characteristics could be observed for the adapted feedstock compositions with capric acid (Figure 13c). They showed considerably lower storage modulus values. This can be seen for the feedstock with 50 vol.% PEG, where the storage modulus for the adapted feedstock at 25 °C is 300 MPa lower than that of its counterpart. The two inhomogeneous feedstocks with the PMMA granulate residues were not measured. The reason for this behavior is due to the higher shear rate and the smaller PMMA granules, which results in more intensive and faster blending of the additive and the polymer chains. On the other hand, the increase of PEG has a negative impact on the flexibility of the filaments. This leads to the conclusion that only the backbone polymers interact with the additives.

#### 3.4.3. Fused Filament Fabrication

Apart from the feedstocks with stearic acid, where the stiff filament rods had to be fed into extruder one by one, all filaments were printed from a spool. As expected, the use of PMMA prevented the filaments from being grounded by the gears of the extruder, yet all materials could be printed. However, the materials with 15 wt.% capric acid and lauric acid showed slight signs of filament abrasion on the gears due to their low hardness. Table 9 lists the investigated printing parameters. As with the PMMA/PEG feedstocks, a nozzle size of 0.15 could be used to print small parts with a high level of detail. However, the materials also exhibited lower green body stability, though this was improved by the influence of the PVB. The use of the part surface ventilation was mandatory and did not lead to clogging in the nozzle despite the high PVB content.

#### 3.4.4. Debinding and Sintering

**Figure 14:**
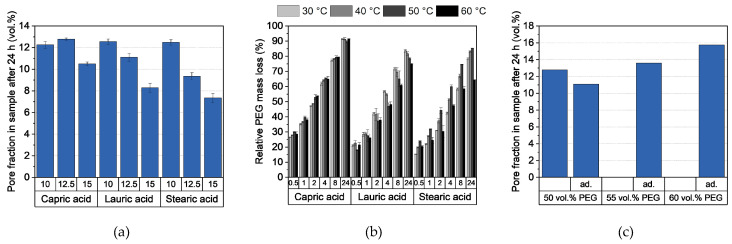
(**a**) Pore fraction in samples with varied fatty acid content after 24 h at 30 °C, (**b**) relative PEG mass loss of feedstocks with 12.5 wt.% additive at different temperatures after 0.5 h, 1 h, 2 h, 4 h, 8 h, and 24 h, and (**c**) pore fraction in samples of feedstocks with varied PEG content and 12.5 wt.% capric acid and in comparison to the adapted (ad.) feedstocks after 24 h at 30 °C. The two inhomogeneous feedstocks with the PMMA granulate residues were not characterized.

Through the high additive content in the feedstocks, the pore fraction in the samples remained below 13 vol.% after 24 h in water at 30 °C (Figure 14a). It is also apparent that debinding degree decreases with increasing additive content. In addition, a noteworthy observation is the decreasing degree of debinding with larger additive molecules for higher additive contents. Possible reasons for this include an increased entanglement between the chains and the slightly higher solubility in water for shorter, more polar chains. Despite the expected diffusion rate being higher at elevated water temperatures, no significant differences in the PEG elimination could be observed after 24 h (Figure 14b). Only in the samples with lauric acid and stearic acid did the degree of debinding begin to decrease at temperatures around 20 °C below their melting point. Figure 14c shows the pore fraction for samples with higher PEG content. As expected, a higher PEG content leads to a higher degree of debinding. Interestingly, this value was reduced in the adapted feedstock compositions. This is likely due to the better homogenization of the feedstocks as a result of the smaller PMMA granules and the higher shear rate during compounding. This led to increased entanglement of the polymer chains which reduced the diffusion of the PEG molecules. With regard to green body strength during solvent debinding, a low PMMA content in the feedstocks ensured sufficient green body strength and the successful manufacturing of parts with complex geometries.

**Figure 15:**
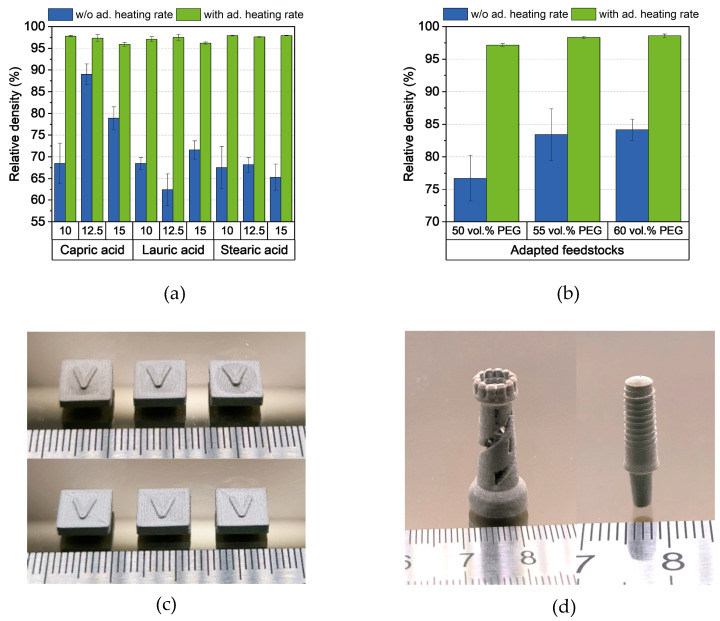
(**a**) Relative density of the samples after sintering without and with the adapted (ad.) heating rate of 0.2 K/min until 250 °C is reached, (**b**) relative density of the samples made from the adapted feedstocks after sintering without and with the adapted heating rate of 0.2 K/min until 250 °C, (**c**) difference of the sintered samples without (upper picture) and with (bottom picture) the adapted heating rate, and (**d**) printed and sintered chess rook and dental implant.

Due to the low pore fraction in the samples, the formation of blisters could be observed in all samples and thus, they each had relative densities below 90% (Figure 15a). The samples made of the adapted feedstocks with higher PEG content showed the same behavior (Figure 15b). Therefore, the heating rate for thermal debinding below 250 °C was set to 0.2 K/min. The temperature was held at 250 °C for 4 h. The standard program for thermal debinding and sintering was continued afterwards. In this case, high relative densities above 97% could be achieved for almost all materials. Only the two feedstocks with 15 wt.% capric acid and lauric acid were found to have lower values, which is likely due to complications occurring during extrusion. The highest relative density of 98.9% was observed for the adapted feedstock with 60 vol.% PEG. This value stands out because there is no record of higher relative density for printed metals without additional hot isostatic pressing in literature [27,49,50]. It even competes with and surpasses metal injection molded parts [18,51,52].

## 4. Conclusions and Outlook

The influence of various additives on the different partly water-soluble binder systems regarding the flexibility and properties of the final parts was investigated. The results indicated that capric acid as well as lauric acid increased the flexibility of PVB-based and PMMA-based filaments. The combination of the two backbone polymers made it possible to produce filaments with high usability and superior properties, such as rapid solidification during cooling and sufficient green body strength during solvent debinding. The use of water instead of organic solvents led to a reduction in cost and potential hazards for both people and the environment, allowing for sustainable part fabrication. With some modifications including a higher PEG content and an adjusted heating rate during thermal debinding, components with relative densities up to 98.9% were manufactured, exceeding all reported density values for this production method.

Furthermore, a method using dynamic mechanical analysis to quantify the flexibility of filaments was introduced and successfully applied for the first time. However, further tests with different fillers and binder systems are required to validate this method. In addition, future research will focus on optimizing debinding and sintering to enable a faster and consequently more cost-effective process. This could be achieved through a higher PEG content in the feedstocks. Possible complications with a corresponding reduction in the hardness of the filaments could be solved by using a dual gear extruder. The use of different and more effective additives could also be an option. An application of the knowledge gained from this study to other fillers such as ceramics and other metals is also planned for future studies.

## Figures and Tables

**Table 1:** Overview of the investigated binder compositions.

Combination	Backbone Polymer	Base Polymer	Additive
PVB/PEG	PVB 30H	PEG 20,000	Capric acid, Lauric acid, Stearic acid
PMMA/PEG	PMMA G7E	PEG 8000	Capric acid, Lauric acid, PAT 77/P
PMMA/PVB/PEG	PMMA G7E/PVB 30H	PEG 8000	Capric acid, Lauric acid, Stearic acid

**Table 2:** Particle properties.

Ti-6Al-4V	Density (g/cm^3^)	D_10_ (µm)	D_50_ (µm)	D_90_ (µm)	SSA (m^2^/g)
Measured	4.4	13.2	28.6	47.3	0.15

**Table 3:** Properties of the used binder components (T_S_: softening temperature, T_D_: decomposition temperature).

Component	Density (g/cm^3^)	M_W_ (g/mol)	T_S_ (°C)	T_D_ (°C)
PMMA G7E	1.18	159,000 ^V^	106	252
PVB 30H	1.11	32,000–35,000 ^V^	64	286
PEG 8000	1.22	7300–9000 ^V^	66	343
PEG 20,000	1.22	20,000 ^V^	69	372
Capric acid	0.89	172 ^V^	36	162
Lauric acid	0.95	200 ^V^	50	184
Stearic acid	0.92	284 ^V^	69	235
PAT-77/P	0.97	n.a.	98	252

^V^: Vendor’s data sheet.

**Table 4:** Extrusion temperatures of the used feedstocks (in °C). The windable filaments are marked.

Additive Content	Capric Acid	Lauric Acid	Stearic Acid
5	100	105	95
7.5	100	100	95
10	105	100	95
20	100	85	95

**Table 5:** Investigated printing parameters for feedstock systems containing 60 vol.% Ti-6Al-4V and a binder composition of 50 vol.% PVB/50 Vol.% PEG 20,000.

Printing Parameter	Capric Acid	Lauric Acid	Stearic Acid
Extrusion temperature (°C)	180–190	180	180
Platform temperature (°C)	60	60	60
Printing speed (mm/s)	8	8	8
Used nozzle diameter (mm)	0.4	0.25–0.8	0.4

**Table 6:** Extrusion temperatures (in °C) and nozzle diameters (in mm) of the feedstocks. The windable filaments are marked.

Additive Content	Capric Acid	Lauric Acid	PAT-77/P
5	50 °C/2.8 mm	55 °C/2.7 mm	72 °C/2.8 mm
7.5	50 °C/2.8 mm	55 °C/2.7 mm	72 °C/2.8 mm
10	47 °C/2.8 mm	55 °C/2.7 mm	70 °C/2.8 mm
20	55 °C/2.8 mm	82 °C/2.8 mm	95 °C/2.8 mm

**Table 7:** Investigated printing parameters for feedstock systems containing 60 vol.% Ti-6Al-4V and a binder composition of 40 vol.% PMMA/60 vol.% PEG 8000.

Printing Parameter	Capric Acid	Lauric Acid	PAT77/P
Extrusion temperature (°C)	190	180	180
Platform temperature (°C)	40	40	40
Printing speed (mm/s)	8	8	8
Used nozzle diameter (mm)	0.4	0.4	0.15–0.4

**Table 8:** Extrusion temperatures (in °C) and nozzle diameters used (in mm) for the feedstocks. The windable filaments are marked.

Additive and PEG Content	Capric Acid	Lauric Acid	Stearic Acid
10 wt.%/50 vol.% PEG	63 °C/2.8 mm	65 °C/2.7 mm	70 °C/2.7 mm
12.5 wt.%/50 vol.% PEG	62 °C/2.7 mm	65 °C/2.7 mm	90 °C/2.8 mm
12.5 wt.%/55 vol.% PEG	52 °C/2.8 mm	-	-
12.5 wt.%/60 vol.% PEG	65 °C/2.7 mm	-	-
15 wt.%/50 vol.% PEG	90 °C/2.8 mm	50 °C/2.8 mm	65 °C/2.8 mm

**Table 9:** Investigated printing parameters for feedstock systems containing 60 vol.% Ti-6Al-4V and a binder composition of 15 vol.% PMMA/35 vol.% PVB/50 vol.% PEG 8000.

Printing Parameter	Capric Acid	Lauric Acid	Stearic Acid
Extrusion temperature (°C)	180–200	190	190
Platform temperature (°C)	60	60	60
Printing speed (mm/s)	2.5–20	8	8
Used nozzle diameter (mm)	0.15–0.8	0.4	0.4

## Data Availability

The data presented in this study are available on request from the corresponding author due to legal issues.
